# Impact of anatomical placement of an accelerometer on prediction of physical activity energy expenditure in lower-limb amputees

**DOI:** 10.1371/journal.pone.0185731

**Published:** 2017-10-05

**Authors:** Peter Ladlow, Tom E. Nightingale, M. Polly McGuigan, Alexander N. Bennett, Rhodri Phillip, James L. J. Bilzon

**Affiliations:** 1 Department for Health, University of Bath, Bath, United Kingdom; 2 Academic Department of Military Rehabilitation, Defence Medical Rehabilitation Centre (DMRC) Headley Court, Surrey, United Kingdom; 3 National Heart and Lung Institute, Faculty of Medicine, Imperial College London, London, United Kingdom; Northwestern University, UNITED STATES

## Abstract

**Purpose:**

To assess the influence of the anatomical placement of a tri-axial accelerometer on the prediction of physical activity energy expenditure (PAEE) in traumatic lower-limb amputees during walking and to develop valid population-specific prediction algorithms.

**Methods:**

Thirty participants, consisting of unilateral (n = 10), and bilateral (n = 10) amputees, and non-injured controls (n = 10) volunteered to complete eight activities; resting in a supine position, walking on a flat (0.48, 0.67, 0.89, 1.12, 1.34 m.s^-1^) and an inclined (3 and 5% gradient at 0.89 m.s^-1^) treadmill. During each task, expired gases were collected and an Actigraph GT3X+ accelerometer was worn on the right hip, left hip and lumbar spine. Linear regression analyses were conducted between outputs from each accelerometer site and criterion PAEE (indirect calorimetry). Mean bias ± 95% limits of agreement were calculated. Additional covariates were incorporated to assess whether they improved the prediction accuracy of regression models. Subsequent mean absolute error statistics were calculated for the derived models at all sites using a leave-one out cross-validation analysis.

**Results:**

Predicted PAEE at each anatomical location was significantly (*P*< 0.01) correlated with criterion PAEE (P<0.01). Wearing the GT3X+ on the shortest residual limb demonstrates the strongest correlation (unilateral; *r* = 0.86, bilateral; *r* = 0.94), smallest ±95% limits of agreement (unilateral; ±2.15, bilateral ±1.99 kcal·min^-1^) and least absolute percentage error (unilateral; 22±17%, bilateral 17±14%) to criterion PAEE.

**Conclusions:**

We have developed accurate PAEE population specific prediction models in lower-limb amputees using an ActiGraph GT3X+ accelerometer. Of the 3 anatomical locations considered, wearing the accelerometer on the side of the shortest residual limb provides the most accurate prediction of PAEE with the least error in unilateral and bilateral traumatic lower-limb amputees.

## Introduction

There is a paucity of research investigating the impact of regular physical activity (PA) on the health and well-being of individuals following recovery from traumatic lower-limb amputation. This is despite considerable evidence from longitudinal cohort studies demonstrating a substantially increased risk of this population developing a range of chronic degenerative disease, including: cardiovascular disease, diabetes, hypertension, obesity and osteoarthritis [1–3].

In order to enhance research and practice in this field, it is important to develop valid and reliable tools to estimate free-living physical activity energy expenditure (PAEE) in lower-limb amputees. This has proven inherently difficult to measure, even in humans without mobility-related physical impairments. This becomes more challenging within a heterogeneous group of individuals with lower-limb loss where the level of amputation results in a varying loss of articular structures and sensory/motor function of the lower-extremity [4]. Indeed, recent investigations into the daily PA and heart rate responses of people with vascular [5] and traumatic [6] unilateral trans-tibial amputations have demonstrated that both amputee groups are less active than matched controls without known physical impairments. These studies highlight the impact of physical disability and associated mobility restriction on volitional PA behaviour.

Despite these investigations, relatively little is known regarding the specific components or patterns of PA that are required to derive protection from chronic diseases and improve metabolic health in these specific population groups. Hence the ability to accurately measure and predict PAEE is critically important in the long-term management and prevention of chronic diseases in persons with lower-limb amputation.

To date, the ability to accurately predict free-living physical activity energy expenditure (PAEE) in unilateral and/or bilateral lower-limb amputees has not been explored. An objective method for assessing habitual PA in this population would allow the development of bespoke PA guidelines, allow appropriate cross-sectional comparisons and enhance research efforts on the efficacy of PA interventions. Previous research on amputee mobility has relied on subjective amputee specific self-reported questionnaires [7–9] in conjunction with objective measures of functional mobility (e.g. step count and timed up and go).

Over 15 years ago, Bussmann and colleagues [10–11] validated the uni-axial IC-3031 and ADX202 body fixed accelerometer in unilateral amputees. However, the aim of these two investigations was only to assess the accelerometers ability to identify posture and motion by comparing outputs to video recordings. Although useful for identifying movements performed in controlled rehabilitation settings, these studies did not attempt to predict energy expenditure, nor the metabolic cost of prosthetic ambulation. Accelerometers are now more widely used in the assessment of human energy expenditure [12–13] and have been shown to be best placed on hip or the lower back during free living activity [14]. Hip based uni-axial and tri-axial accelerometers have been used for the measurement of PA in clinical populations with functional limitations including: stroke [15]; multiple sclerosis and; Parkinson’s disease [16]. To date, there are no published studies to determine the most appropriate anatomical placement of accelerometers to accurately predict PAEE in unilateral or bilateral amputees. Consequently, there are no peer-reviewed articles, which have attempted to develop population specific algorithms for the prediction of PAEE in lower-limb amputees.

Recent technological advancements in the field of PAEE measurement has stimulated the development of sensitive tri-axial accelerometers, which are unobtrusive, low-cost, capable of storing higher resolution raw, unfiltered acceleration signals over prolonged periods of time [17]. The Actigraph^™^ GT3X+, is a tri-axial accelerometer that has previously been validated in wheelchair users [18]. The ability of the GT3X+ accelerometer to accurately predict PAEE over a variety of ambulatory velocities and gradients in people with unilateral and bilateral lower-limb amputation remains unclear. Due to differences in ambulatory efficiency between unilateral and bilateral amputees compared to able-bodied adults, amputee specific PAEE prediction models should be determined. This study aimed to evaluate the effect of anatomical positioning around the pelvis of the GT3X+ accelerometer on the prediction of PAEE in unilateral and bilateral amputees and to develop population specific algorithms for a range of ambulatory velocities in a controlled-laboratory environment.

## Materials and methods

Ethics approval was granted by the United Kingdom Ministry of Defence Research Ethics Committee (MODREC) and written informed consent was obtained from each participant. A convenient sample of ten unilateral and ten bilateral military amputees and ten non-injured healthy controls volunteered to participate in this study. All participants were male and visited the Military Performance and Rehabilitation Laboratory (MPARL) at the Defence Medical Rehabilitation Centre (DMRC), Headley Court on one morning after a ten hour overnight fast. Inclusion criteria included all injured participants having experienced traumatic amputation and had previously received at least three 4-week admissions of intensive exercise rehabilitation at DMRC Headley Court from an interdisciplinary team of health professionals [19]. All patients received a prosthetic fitting prior to commencing the trial and had been given clearance to ambulate on a treadmill by their physiotherapist. Exclusion criteria were based upon the participant’s medical history (screened by their physician). This includes severe traumatic brain injury, medication that alters heart rate variability, and any mobility restricting conditions, such as painful heterotopic ossification or insufficient wound healing around the stump. The control group are non-injured physically active men (civilian and military who engage in aerobic or resistance based training at least three times per week) employed by the MOD.

### Accelerometer

Throughout the activity protocol, three GT3X+ units were worn, one on either side of the waist, above the hip (along the anterior axillary line) and one on the lower back (positioned on L2) all using an elasticated belt. Following the Nyquist principle, the devices were initialised with a sampling frequency of 30 Hz, thereby allowing the capture of general human movement [20]. The componentry and capabilities of the Actigraph GT3X+ has previously been reported [18].

### Indirect calorimetry

Participants wore a facemask connected to a portable metabolic system (Metamax 3B, Cortex, Leipzig, Germany) and expired gases were collected throughout each activity protocol. Metabolic data were retrieved and analysed using the Metamax software. Oxygen uptake (${\dot{\text{V}}\text{O}}_{2}$) and carbon dioxide production (${\dot{\text{V}}\text{CO}}_{2}$) were used to estimate ‘steady-state’ energy expenditure (kcal·min^-1^) of each activity using indirect calorimetry. Before use, the Metamax was calibrated according to manufacturer’s instructions.

### Test protocol

#### Phase 1

Anthropometric data were collected at the start of the protocol, including: body mass (with and without prosthesis), stature, hip and waist circumference and, level and number of amputations (below knee, through knee and above knee). The time since amputation, which indicates the length of rehabilitation (months), was also recorded. All amputations were performed at an anatomical level above the ankle and below the hip. The Metamax 3B and the three GT3X+ activity monitors were synchronised before use. Resting metabolic rate (RMR; kcal·day^-1^) was measured in a semi-recumbent position in accordance with best practice guidelines [21]. Following the measurement of RMR and anthropometric assessment, participants completed a walking protocol on a level treadmill (Woodway Desmo, USA). This protocol consisted of ambulating at 5 progressive velocities (0.48, 0.67, 0.89, 1.12, 1.34 m.s^-1^ or 1, 1.5, 2, 2.5 and 3 mph, respectively) and 2 gradients (3% and 5%) at 0.89 m.s^-1^ (2 mph). The velocities were determined by self-selected walking speeds performed on a similar group of UK military amputees [22]. This would provide a wide range of ambulatory velocities to be captured in a heterogeneous group of amputees. Each activity lasted 5 minutes with no recovery between each intensity increments. Participants were asked to complete the entire protocol without resting their arms on the handrail. Participants were told to stop if they experience residuum pain, prosthetic discomfort or difficulty maintaining the speed of the treadmill belt to a point where they felt they were at risk of falling. Rating of perceived exertion (RPE) was collected at the end of each treadmill intensity using the 6–20 Borg Scale [23].

#### Phase 2

Although standing and ambulatory tasks form the primary basis of amputee rehabilitation, sitting based arm-exercises are sometimes utilised in the rehabilitation environment. Therefore, participants also performed sitting-based arm crank ergometry (ACE) (Technogym Excite, UK) after the cessation of phase 1, at three different cadences (50, 70, 90 rpm) with a fixed resistance (55 W). This enabled us to test whether the ambulatory predictive equations were capable of accurately predicting PAEE during seated arm-exercise.

### Calculating PAEE

Breath-by-breath data was exported into Microsoft Excel from the Metamax 3B. PAEE was then calculated using the ${\dot{\text{V}}\text{O}}_{2}$ and ${\dot{\text{V}}\text{CO}}_{2}$ values (l·min^-1^) from the Metamax in an Excel spreadsheet using the Weir equation [24]. Assuming that dietary-induced thermogenesis was negligible (participants came into the laboratory following a 10 hour overnight fast) resting metabolic rate (kcal·min^-1^) was subtracted from total energy expenditure to determine PAEE. Metabolic equivalent (METs) were then calculated using measured exercise ${\dot{\text{V}}\text{O}}_{2}$ divided by resting ${\dot{\text{V}}\text{O}}_{2}$ to derive individual METs in the last 2 minutes of each treadmill intensity. Comparisons between accelerometer outputs (PAC) and criterion PAEE were made between the final two-minutes of each activity (representative of steady-state).

The GT3X+ accelerometer units were downloaded using ActiLife software (ActiGraph, Pensocola, FL, USA). Data was exported to Microsoft Excel in a time and date stamped comma-separated value (CSV) file format. Activity counts (counts·min^-1^) from the GT3X+ were then averaged over the corresponding final two minutes of each activity.

### Statistical analyses

PAEE prediction models were developed using corresponding data from each task for devices at each location, using linear regression analysis. The dependent variable was PAEE (kcal·min^-1^) during the final 2 minutes of each task (that is 80 values in each group). The independent variable was accelerometer outputs (counts·min^-1^) for the GT3X+. Pearson product moment correlation coefficients (*r*) and coefficients of determination (R^2^) statistics were reported to assess the association between criterion PAEE and outputs from devices at each location. Standard Error of the Estimate (SEE) was calculated for each model (Model 1).

The GT3X+ worn at the anatomical position with the strongest relationship to the criterion PAEE was then selected for further analysis, to develop a predictive model for PAEE. Covariates, which included age, body mass, waist circumference, time since amputation, and level of amputation, were analysed to determine their association with the criterion PAEE depending on if data was discrete or continuous. These covariates were selected due to their influence upon mobility in US military amputees [25]. Significant covariates were included in the stepwise regression analysis to strengthen the predictive PAEE equations in each group (Model 2).

These predictive models should be cross-validated using an independent sample. However, this is not always possible in hard to reach populations due to recruitment issues. To overcome this problem prediction algorithms, to determine the PAEE prediction error, were developed using a systematic ‘leave-one-out’ cross validation analysis [26], as performed previously by Nightingale *et al*. [27]. In summary, this process was repeated where each participant acted as the ‘held-out’ participant and the mean error of all calculations was determined. Error statistics involved calculating the mean absolute error, mean absolute percentage error and mean signed error for each activity; the latter displayed graphically using Bland and Altman plots and limits of agreement analysis. A two way mixed model ANOVA was performed to determine differences between criterion PAEE and predicted PAEE at each treadmill task. Where a significant interaction effect was observed, a Bonferroni correction was applied to Post Hoc tests where multiple comparisons were considered. This was to identify the specific treadmill tasks in which there was a significant difference between the criterion and predicted PAEE. Statistical significance was set a priori of *P* < 0.05. All analysis was performed using IBM SPSS Statistics 21 for Windows (IBM, NY, USA).

## Results

Demographic and physical characteristics of the participants are described in Table 1. Criterion PAEE (kcal·min^-1^), ActiGraph GT3X+ accelerometer outputs at each anatomical location, RPE and METs are displayed in Table 2. Not all amputee participants were able to complete all of the treadmill speeds in this trial. The number of participants that dropped out of each treadmill task is also presented in Table 2. Despite the lower mean RMR values in the bilateral amputee group, there were no significant main effects or group differences (p>0.05). There was a significant main effect on actual PAEE, predicted PAEE, and METs, with significant differences between all three groups. Mean PAEE, PAC, RPE and METs increased with increasing velocity of the treadmill in the unilateral and control group. In the bilateral group, six to eight participants were unable to complete activities at the higher treadmill velocities (see Table 2).

**Table 1:** Demographic and physical characteristics of the participants. Information displayed as mean ± SD.

Variable	Unilateral		Bilateral		Control	
	Mean ± SD	Range	Mean ± SD	Range	Mean ± SD	Range
Number of Participants	10		10		10	
Age (years)	32 ± 5	23–41	29 ± 4	22–34	32 ± 6	25–45
Body Mass—without prosthesis (kg)	81 ± 11	63–108	82 ± 19	59–126	79 ± 7	68–89
Waist Circumference (cm) *	92 ± 12	75–115	100 ± 20	77–149	84 ± 4	76–90
Waist-hip ratio	0.90 ± 0.06	0.83–1.00	0.94 ± 0.09	0.86–1.17	0.86 ± 0.04	0.79–0.92
RMR (kcal·d^-1^)	1800 ± 264	1480–2158	1596 ± 178	1382–2051	1808 ± 217	1463–2059
Time Since Amputation (months) ǂ	24 ± 15	4–46	39 ± 14	21–61	-	-
*Level of Amputation*:						
Below Knee	6		1		-	
Through Knee	2		2		-	
Above knee	2		3		-	
Bilateral: Below Knee and Above Knee	-		2		-	
Bilateral: Through Knee and Above Knee	-		2		-	

*Significant difference between bilateral amputees and control group (*P*<0.05).

^ǂ^ Significant difference between unilateral and bilateral amputees (*P*<0.05).

**Table 2:** Measured PAEE, accelerometer outputs at each anatomical location, calculated METs, RPE and number of participants for each activity (mean ± SD).

Activity	PAEE Metamax 3B (kcal·min^-1^)	GT3X+ (PAC·min^-1^)			METS (calculated)	RPE	n
		Longest Limb	Spine	Shortest Limb			
**Unilateral Amputees:** ǂ # ¥							
**RMR**	0.0 ± 0.0	0 ± 0	0 ± 0	0 ± 0	1.0 ± 0.0	6 ± 0	10
**Treadmill 0.48 m.s**^**-1**^	2.4 ± 0.7	2361 ± 710	2256 ± 777	2691 ± 831	3.1 ± 0.7	8 ± 1	10
**Treadmill 0.67 m.s**^**-1**^	2.9 ± 0.9	2665 ± 639	2517 ± 577	2945 ± 856	3.5 ± 0.9	9 ± 1	10
**Treadmill 0.89 m.s**^**-1**^	3.6 ± 1.1	3038 ± 583	2904 ± 597	3384 ± 848	4.0 ± 1.0	11 ± 2	10
**Treadmill 1.12 m.s**^**-1**^	4.3 ± 1.4	3723 ± 457	3673 ± 521	4130 ± 670	4.6 ± 1.3	12 ± 2	10
**Treadmill 1.34 m.s**^**-1**^	5.6 ± 1.7	4703 ± 674	4794 ± 660	5126 ± 539	5.3 ± 1.6	12 ± 2	7
**Treadmill 3% (0.89 m.s**^**-1**^**)**	4.1 ± 1.1	3131 ± 514	3044 ± 648	3671 ± 929	4.4 ± 1.1	11 ± 1	10
**Treadmill 5% (0.89 m.s**^**-1**^**)**	4.8 ± 1.2	3370 ± 537	3258 ± 492	4018 ± 948	4.9 ± 1.2	12 ± 2	10
Bilateral Amputees:* §							
**RMR**	0.0 ± 0.0	0 ± 0	0 ± 0	0 ± 0	1.0 ± 0.0	6 ± 0	10
**Treadmill 0.48 m.s**^**-1**^	3.7 ± 1.4	4132 ± 1645	3449 ± 696	4800 ± 1410	4.4 ± 1.2	10 ± 2	10
**Treadmill 0.67 m.s**^**-1**^	4.6 ± 1.5	4453 ± 2044	3792 ± 829	5264 ± 1603	5.1 ± 1.4	12 ± 2	10
**Treadmill 0.89 m.s**^**-1**^	5.5 ± 1.7	4843 ± 2101	4199 ± 822	5600 ± 1502	5.8 ± 1.6	14 ± 3	10
**Treadmill 1.12 m.s**^**-1**^	5.5 ± 2.9	6596 ± 3943	4846 ± 1363	6123 ± 2823	5.3 ± 1.4	15 ± 3	3
**Treadmill 1.34 m.s**^**-1**^	6.3 ± 2.9	5251 ± 835	4907 ± 518	5235 ± 1212	5.7 ± 1.7	15 ± 0	2
**Treadmill 3% (0.89 m.s**^**-1**^**)**	5.9 ± 2.3	5064 ± 1771	4408 ± 834	5973 ± 1592	6.1 ± 2	14 ± 3	8
**Treadmill 5% (0.89 m.s**^**-1**^**)**	5.8 ± 1.9	5594 ± 2509	4813 ± 1289	5806 ± 2231	5.7 ± 1.1	16 ± 2	4
**Control:**							
**RMR**	0.0 ± 0.0	0 ± 0	0 ± 0	0 ± 0	1.0 ± 0.0	6 ± 0	10
**Treadmill 0.48 m.s**^**-1**^	1.4 ± 0.3	1542 ± 495	1352 ± 455	1416 ± 536	2.2 ± 0.3	7 ± 0	10
**Treadmill 0.67 m.s**^**-1**^	1.8 ± 0.3	2006 ± 336	1776 ± 278	1876 ± 403	2.5 ± 0.3	8 ± 1	10
**Treadmill 0.89 m.s**^**-1**^	2.3 ± 0.4	2577 ± 369	2290 ± 332	2478 ± 459	2.9 ± 0.4	9 ± 1	10
**Treadmill 1.12 m.s**^**-1**^	2.8 ± 0.4	3463 ± 398	3162 ± 394	3353 ± 461	3.3 ± 0.4	9 ± 1	10
**Treadmill 1.34 m.s**^**-1**^	3.3 ± 0.4	4321 ± 469	4096 ± 429	4205 ± 471	3.7 ± 0.3	10 ± 1	10
**Treadmill 3% (0.89 m.s**^**-1**^**)**	2.9 ± 0.4	2732 ± 243	2395 ± 265	2591 ± 385	3.4 ± 0.3	10 ± 1	10
**Treadmill 5% (0.89 m.s**^**-1**^**)**	3.4 ± 0.4	2924 ± 280	2598 ± 187	2766 ± 327	3.8 ± 0.3	10 ± 1	10

*Due to reduced participant numbers, all statistical analyses comparing the bilateral group with other groups were performed at speeds **0.48–0.89 m.s**^**-1**^ and at 3% gradient.

^ǂ^ A significant difference in criterion PAEE and METs were only reported at higher intensities (**1.12 m.s**^**-1**^, **1.34 m.s**^**-1**^ and 5% gradient at **0.89 m.s**^**-1**^) between the unilateral amputees and control group (*P*<0.05).

^§^ A significant differences in criterion PAEE, METs, PAC (GT3X+ worn at the longest and shortest limb) were found between bilateral amputees versus the unilateral and control groups all speeds analysed (*P*<0.05).

^#^ Significant differences in PAC (GT3X+ worn on shortest limb) were only reported at the higher intensities of **1.34 m.s**^**-1**^ and 5% gradient at **0.89 m.s**^**-1**^ between unilateral amputees and control group (P<0.05).

^¥^ Significant difference in PAC (GT3X+ worn on spine) were found at **0.48 m.s**^**-1**^, **0.67 m.s**^**-1**^ and 3% Gradient at **0.89 m.s**^**-1**^ between unilateral amputees and control group (P<0.05).

All participants with a through and/or above knee amputation, in both groups (i.e. unilateral and bilateral), wore a Genium prosthetic device during all activities. Five of the seven below knee amputees wore a Variflex XC, whilst one used an Echelon VT and the other wore a Panthera CF2. The prosthetic devices worn by the bilateral amputees with an above and below knee combination included a BiOM and a Variflex XC for the below knee prosthesis and a Genium for their above knee amputation.

Across all treadmill walking tasks, PAEE was 1.4 to 1.7 times greater in the unilateral amputees compared to the non-injured controls. At treadmill speeds between 0.48 to 0.89 m.s^-1^ and a gradient of 3% at 0.89 m.s^-1^, PAEE was 2 to 2.6 times greater in bilateral amputees compared to controls.

At these same walking speeds PAEE was 1.4 to 1.6 times greater in the bilateral compared to the unilateral amputees. The difference in PAC between both amputee groups versus control was greatest at slower walking speeds with the relative differences in PAC reducing at faster walking speeds. There was a significant difference in PAC (GT3X+ worn on shortest limb) between all groups at 0.48 m.s^-1^, 0.67 m.s^-1^ and 0.89 m.s^-1^ (*P* < 0.001). The MET data suggest that, for all walking speeds above 0.48 m.s^-1^, for the unilateral and bilateral groups, exercise intensity was considered to be of moderate-intensity. For the non-injured control group, moderate intensity activity only occurred at a walking speed of 1.12 m.s^-1^.

**Fig 1:**
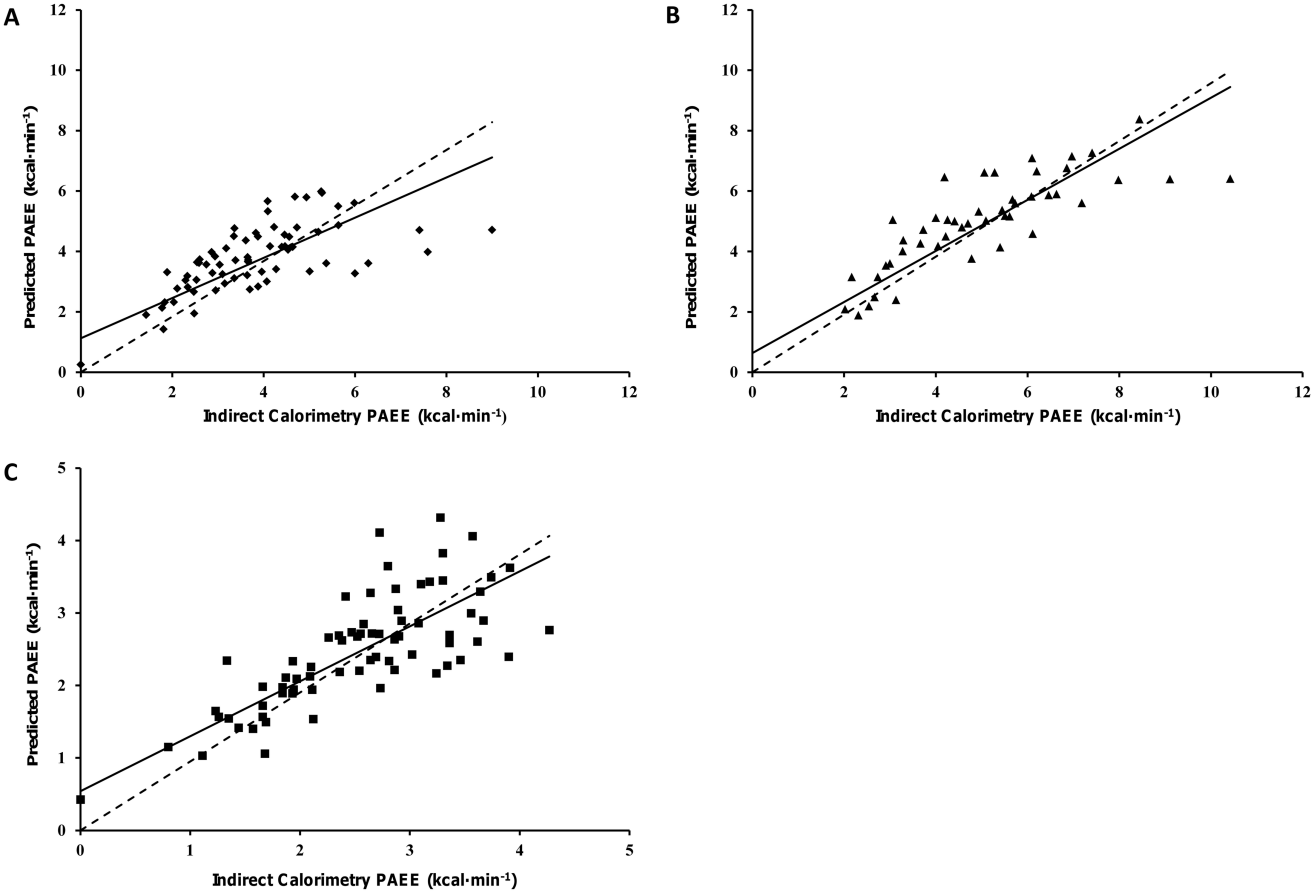
Scatterplots showing the relationship between predicted PAEE for the GT3X+ worn on the hip of the shortest limb and criterion PAEE (Model 1). (A) unilateral amputee group, (B) bilateral amputee group and (C) the left hip of the non-injured control group. The straight line represents the models best fit, and the dotted line indicates the line of identity.

PAC from each anatomical location were significantly (*P*<0.01) associated with criterion PAEE. In both of the amputee groups the GT3X+ worn on the hip with the shortest residual limb demonstrated the strongest relationship, smallest limits of agreement (LoA) (Table 3) and mean absolute error (See S1 Table, which illustrates the error of the GT3X+ monitor at each anatomical location and the generated predictive model for all three groups). In the control group, the strength of the relationship and level of error was similar at each anatomical location. The correlation between criterion and predicted PAEE at the most accurate anatomical location, for each group, are presented in Fig 1a–1c.

**Table 3:** The relationship between predicted PAEE using the Actigraph GT3X+ and criterion PAEE at the three anatomical positions in all three groups.

Location	r	R^2^	SEE (kcal·min^-1^)	LoA (kcal·min^-1^)	P Value
**Unilateral Amputee Group**					
Longest Residual Limb	0.76	0.59	1.23	0 ± 2.39	<0.001
Spine	0.68	0.46	1.40	0 ± 2.73	<0.001
Shortest Residual Limb	0.82	0.67	1.11	0 ± 2.15	<0.001
**Model 1.1:** PAEE = (0.000979 x PAC·min^-1^) + 2.255481					
**Model 2.1:** PAEE = (0.000928 x PAC·min^-1^) + (0.027761 x Time Since Amputation[months]) + (0.663267 x Level of Injury [1 or 2]) − 1.139788					
Shortest Residual Limb	0.86	0.73	1.01	0 ± 1.91	<0.001
**Bilateral Amputee Group**					
Longest Residual Limb	0.80	0.64	1.56	0 ± 3.03	<0.001
Spine	0.80	0.64	1.57	0 ± 3.05	<0.001
Shortest Residual Limb	0.92	0.85	1.03	0 ± 1.99	<0.001
**Model 1.2:** PAEE = (0.000929 x PAC·min^-1^) − 0.051541					
**Model 2.2:** PAEE = (0.000877 x PAC·min^-1^) + (0.024560 x Waist Circumference [cm]) − 2.263715					
Shortest Residual Limb	0.94	0.88	0.93	0 ± 1.79	<0.001
**Control Group**					
Left Limb	0.88	0.77	0.54	0 ± 1.06	<0.001
Spine	0.87	0.75	0.57	0 ± 1.10	<0.001
Right Limb	0.87	0.76	0.56	0 ± 1.08	<0.001
**Model 1.3:** PAEE = (0.000776 x PAC·min^-1^) + 0.427097					
**Model 2.3:** PAEE = (0.000782 x PAC·min^-1^) + (0.033104 x Body Mass [kg]) − 2.191630					
Left Limb	0.89	0.80	0.51	0 ± 0.98	<0.001

The table displays the predictive equations used in the most accurate accelerometer location, the shortest residual limb (Model 1) and the impact of significant covariates (Model 2) at increasing the accuracy of the GT3X+ accelerometer at predicting PAEE. Limits of agreement (LoA) expressed as mean ± 95% SD.

**Fig 2:**
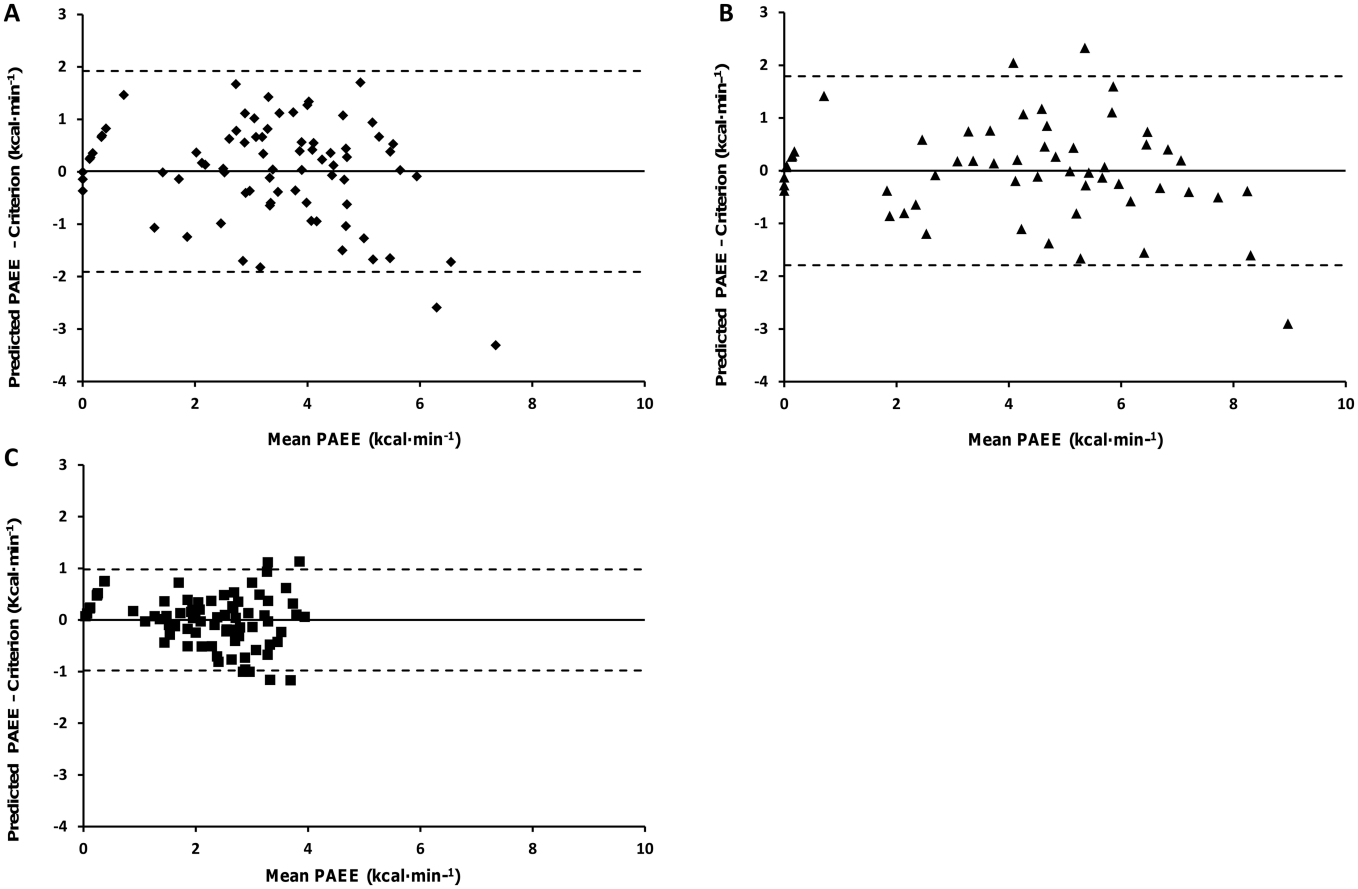
Bland and Altman plots for the criterion and predicted PAEE using cross-validated, population specific prediction models (Model 2). Developed for the unilateral group (A), bilateral group (B) and control group (C). The straight line demonstrates the mean and the dotted line indicates the 95% Limits of Agreement (LoA).

**Fig 3:**
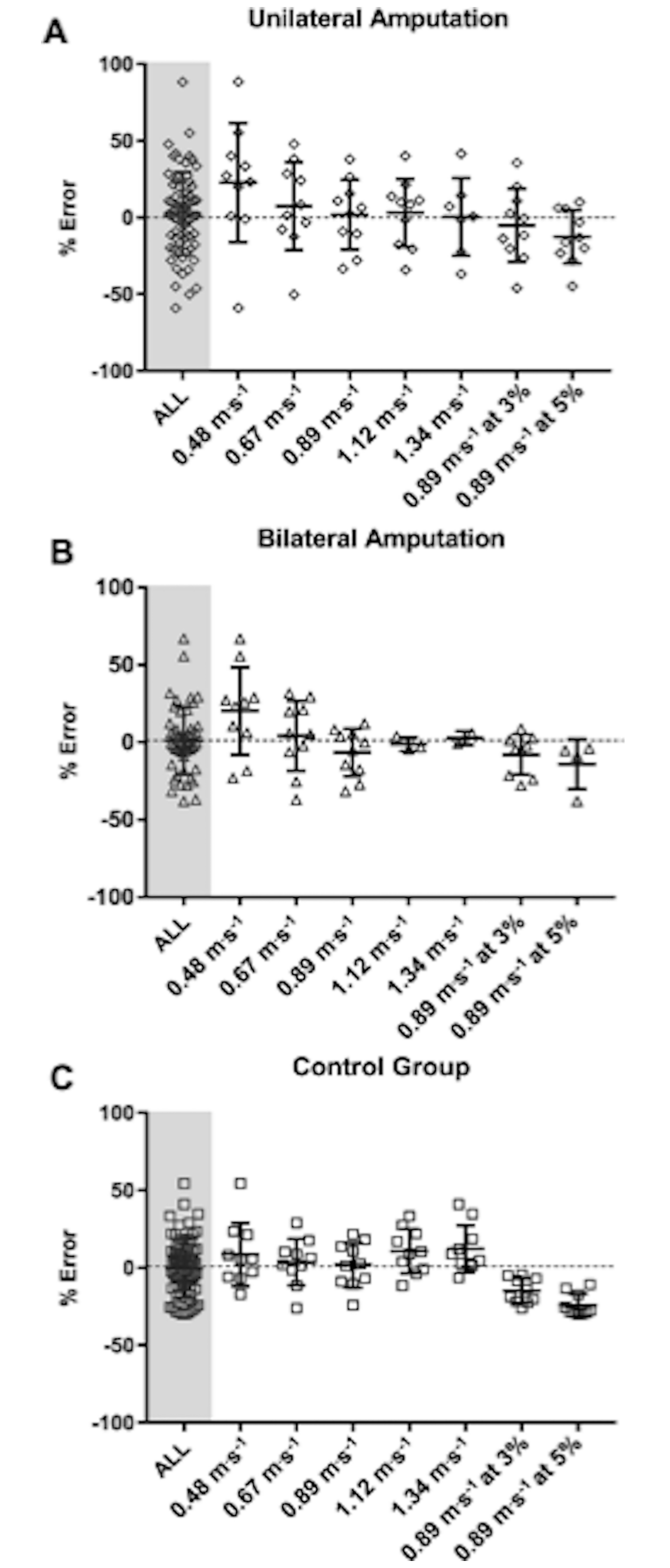
Modified box and whisker plots demonstrating the mean percentage error of estimation relative to criterion for each treadmill activity using the cross validated, population specific prediction models (Model 2). The plots show the unilateral group (A), bilateral group (B) and control group (C).

Fig 2 (panels a-c) illustrates the difference between criterion and predicted PAEE derived from population specific prediction models (Model 2) through the use of Bland and Altman plots [mean ± 95% limits of agreement (LoA)]. These reveal a degree of heteroscedasticity (error increases as exercise intensity increases) in the control group. When comparing the two amputee groups these plots demonstrate similar mean bias between groups, which is greater than that in the control group. There are considerably larger LoA in the bilateral amputee group across all treadmill tasks. Mean absolute error statistics between the criterion and estimated PAEE from each anatomical location and the generated model for each treadmill task are shown in the Supplemental Table 1. Greatest error was reported in the unilateral amputee group (mean absolute percentage error, unilateral: 21±17%, bilateral: 16±15%, control: 15±7%) using the population specific generated models. Modified box and whisker plots depicting the mean percentage error of estimation relative to criterion for each treadmill activity using the cross validated, population specific prediction models (model 2) are found in Fig 3.

When these generated regression equations were applied to ACE they were not significantly associated with the criterion PAEE in the unilateral and control group (*P* > 0.05) and demonstrated a weak correlation (unilateral: *r* = 0.32, R^2^ = 0.11, SEE = 0.72 kcal·min^-1^, bilateral: *r* = 0.68, R^2^ = 0.47, SEE = 0.42 kcal·min^-1^, control: *r* = 0.21, R^2^ = 0.04, SEE = 1.06 kcal·min^-1^). The equations also significantly under-predict PAEE; absolute mean bias ± 95% LoA were -2.81 ± 1.52 kcal·min^-1^, -3.08 ± 0.92 kcal·min^-1^ and -2.88 ± 1.06 kcal·min^-1^ for the unilateral, bilateral and control group, respectively.

## Discussion

To our knowledge, this is the first accelerometer-based validation study to predict PAEE in lower-limb amputees. This is the first controlled-laboratory trial to determine the most appropriate anatomical wear location for a tri-axial accelerometer and cross-validate population specific (both unilateral and bilateral amputees) PAEE prediction models across a range of ambulatory velocities. Our results demonstrate that the anatomical position of accelerometers is an important consideration when assessing PAEE in people with lower-limb amputations and, potentially, other functional limitations. Of the three anatomical locations considered in this study, the results indicate that the GT3X+ worn on the hip of the shortest residual limb elicits the strongest correlations with criterion PAEE, explaining the greatest amount of variance and displayed the lowest random error in traumatic lower-limb amputees. The population specific prediction models, which were cross-validated, explain 73%, 88% and 80% of the variability in PAEE measurement in the unilateral, bilateral and control groups, respectively.

One explanation for the lower random error associated with wearing the GT3X+ accelerometer on the shortest residual limb, as opposed to the spine or longest residual limb, is likely due to the increased ability to detect atypical movements (PAC) on that side of the body. It was previously reported that pelvic range of motion in the frontal plane is typically increased in lower-limb amputees compared to able-bodied controls [28]. Amputees often lift their pelvis on the swing side while walking. This compensatory motion, known as hip hiking, is often seen in both trans-tibial and trans-femoral amputees and is believed to compensate for the inability to produce dorsi-flexion in the prosthetic ankle [29]. Hip hiking increases the prosthetic foot clearance [28], but may also be associated with additional metabolic cost of raising the body centre of mass, thus reducing gait efficiency. Individuals with bilateral amputations may display bilateral hip hiking on both sides, which appears to further increase the energy cost of locomotion [29]. The increase of hip hiking and exaggerated movements on the shortest residual limb may explain the increase in PAC recorded on this GT3X+ accelerometer. However, it was beyond the scope of this study to verify this hypothesis by performing kinematic and/or kinetic analyses of amputee gait. The biomechanics of lower-limb amputee gait have been reviewed elsewhere [29].

Bilateral amputees typically demonstrate lower levels of physical function than unilateral amputees. Therefore, at the highest ambulation velocities, they have higher PAEEs. The stronger correlation in the bilateral amputee cohort in the current study is likely to be an artefact of there being [19] a wider range of functional mobility in this group, which explains the larger range of PAEE. Due to the drop out of participants, only the bilateral amputees who had better ambulatory efficiency were able to perform the higher velocities. This could explain why there was reduced error in the bilateral amputee group compared to the unilateral amputee groups.

The inclusion of the covariates in the predictive models provided small but significant improvements in the variance explained in PAEE (see Table 3). The preservation of the knee joint and the utilisation of the knee and hip musculature when ambulating appear to provide a significant functional advantage in unilateral amputees [30]. This is evident from our regression analysis that found the level of amputation is a significant predictor of PAEE in our unilateral amputee group. Time since amputation was also significantly associated with PAEE in the unilateral amputee group. It appears that the longer the time since limb-loss, the better the efficiency of ambulating with a prosthesis. All amputee patients in this study have been engaged in the Defence Medical Services rehabilitation pathway at DMRC, Headley Court [19] since the point of injury. This suggests that the longer the recovery time in conjunction with access to intensive exercise rehabilitation, the greater the opportunity to maximise physical function and prosthetic use.

Waist circumference was a significant predictor of PAEE in both amputee groups. Greater waist circumference was associated with increased PAEE when walking at a variety of speeds. Although body mass significantly predicted PAEE in the bilateral amputees it was not included in the predictive model due to the stronger influence of the waist circumference measurement on PAEE. One explanation for this is that total body mass is unable to differentiate between lean and adipose tissue, whereas, waist circumference gives an indication of central adiposity, suggesting the excess body mass in the bilateral amputees group may in fact be increased adipose tissue and not lean skeletal muscle tissue that could contribute to greater strength capabilities and increased levels of function. Gaunaurd *et al*. 2013 [25] investigated factors relating to high level mobility in US military servicemen with lower-limb loss and also found these same covariates were predictors of high mobility.

It is advisable not to use the generated equations to predict PAEE during seated arm crank exercise, as the models significantly under-predicted PAEE in all groups and showed particularly weak correlation in the unilateral group. If amputees are wheelchair dependent, or perform most of their exercise rehabilitation or recreational activity in a seated position, a different anatomical position or activity monitor could be recommended. Recent research by Nightingale *et al*.2014 [18] reveals that in wheelchair users the GT3X+ monitor worn at wrist is a stronger predictor of PAEE compared to a GT3X+ being worn at the hip.

One potential limitation of this study was the relatively small sample size and the within-group variations in the severity of lower-limb loss injuries. However, this diversity may be considered beneficial as the range of functional abilities improves the external validity of the regression equations, making them more suitable for the wider amputee population. Also despite the diversity of the population, the amount of unexplained random error is relatively small. The inclusion of a diverse range of participants is in accordance with best practice recommendations for PA validation studies [31]. We recognise that the amputee cohort has received intensive rehabilitation unique to the UK military [19] and this is likely to have an effect on their walking efficiency. As a population group, military personnel are predominantly male, aged 20–40 years of age and have undergone physical training in the course of their career. Although some of the mechanisms of injury (e.g. blast injuries) may be different to a civilian population, the types of injuries sustained by our unilateral cohort are not too dissimilar from what might be expected in road traffic accidents (e.g. motorbike) and some adventure sports. Therefore, we believe that our findings are applicable to the physically active civilian lower-limb amputee population.

It is important to note that participants were not provided with a familiarisation to treadmill walking prior to starting the trial. This may have affected the energy expenditure recorded due to the lack of familiarity with the exercise task, therefore requiring greater effort from the participants. This study did not measure self-selected walking speed over-ground. There may have been a difference in the energy cost of walking over-ground compared with treadmill walking [32], potentially reducing the accuracy of using these generated equations in the assessment of free-living ambulatory tasks. Empirical evidence suggest that civilian bilateral amputees are likely to be reliant on a wheelchair to mobilise during free-living conditions, therefore bilateral amputees from the wider civilian population may be unlikely to complete this treadmill protocol due to the intensity and duration spent in ambulation. We felt the inclusion of bilateral amputees who had undergone intensive rehabilitation was important to allow us to make comparisons with unilateral amputees and non-injured controls. This study design allows greater insight into the energy cost of walking in a wider cohort of severely injured individuals.

Future studies should investigate the utility of the GT3X+ to accurately assess PAEE in free-living conditions. This will allow interventions to be developed that provide greater understanding of the specific components and patterns of PA that are required to derive protection from chronic diseases and improve cardio-metabolic health. Data from other disabled populations demonstrate that multi-sensor devices, which combine accelerometry and heart rate signals, are superior predictors of PAEE than accelerometers alone [33]. It would be useful to establish if the same is true in a lower-limb amputee population, when compared to able-bodied controls.

## Conclusion

Of the three anatomical locations considered, wearing the accelerometer on the side of the shortest residual limb provides the most accurate prediction on PAEE in lower-limb amputees. Provided that the predictive equations incorporate additional covariates to increase the accuracy of the monitor to predict PAEE, the GT3X+ accelerometer could be used as a valid tool for the assessment of free-living PAEE in traumatic lower-limb amputees.

## Supporting information

S1 Table Mean absolute error (MAE); kcal·min^-1^) and mean absolute percentage error of predicted PAEE using generated linear regression equations for each anatomical location and the most accurate ‘generated model’ which uses additional covariates (Model 2). (DOCX)
