# Supplementary material for: Impact of anatomical placement of an accelerometer on prediction of physical activity energy expenditure in lower-limb amputees
Source: PLoS One. 2017 Oct 5;12(10):e0185731. doi: 10.1371/journal.pone.0185731 (PMC5628873; doi:10.1371/journal.pone.0185731)
Supplement: S1 Table — (DOCX) [file pone.0185731.s001.docx]

Supplemental Digital Content Table 1: Mean absolute error (MAE); kcal·min^-1^) and mean absolute percentage error of predicted PAEE using generated linear regression equations for each anatomical location and the most accurate ‘generated model’ which uses additional covariates (Model 2).

| **Activity** | **MAE (kcal·min^-1^)** | | | | | | | | | | | |
| --- | --- | --- | --- | --- | --- | --- | --- | --- | --- | --- | --- | --- |
|  | **Unilateral Amputees** | | | | **Bilateral Amputees** | | | | **Control** | | | |
|  | **Longest Limb** | **Spine** | **Shortest Limb** | **Generated model** | **Longest Limb** | **Spine** | **Shortest Limb** | **Generated model** | **Left Limb** | **Spine** | **Right Limb** | **Generated model** |
| **Resting** | 0.36 ± 0.01 | 0.79 ± 0.00 | 0.26 ± 0.00 | 0.50 ± 0.42 | 0.98 ± 0.00 | 0.24 ± 0.00 | 0.05 ± 0.00 | 0.36 ± 0.39 | 0.34 ± 0.00 | 0.44 ± 0.00 | 0.43 ± 0.00 | 0.41 ± 0.24 |
| **0.48 m.s^-1^** | 0.97 ± 0.51 | 0.98 ± 0.59 | 0.83 ± 0.36 | 0.77 ± 0.55 | 1.18 ± 0.53 | 1.30 ± 1.13 | 1.04 ± 0.65 | 0.99 ± 0.68 | 0.30 ± 0.29 | 0.38 ± 0.22 | 0.32 ± 0.30 | 0.21 ± 0.21 |
| **0.67 m.s^-1^** | 0.78 ± 0.69 | 0.85 ± 0.75 | 0.69 ± 0.50 | 0.62 ± 0.49 | 1.27 ± 0.95 | 1.15 ± 1.28 | 0.91 ± 0.54 | 0.80 ± 0.53 | 0.22 ± 0.20 | 0.21 ± 0.12 | 0.20 ± 0.18 | 0.20 ± 0.15 |
| **0.89 m.s^-1^** | 0.77 ± 0.88 | 0.91 ± 1.03 | 0.74 ± 0.80 | 0.67 ± 0.52 | 1.49 ± 1.25 | 1.19 ± 1.27 | 0.77 ± 0.80 | 0.68 ± 0.57 | 0.28 ± 0.21 | 0.24 ± 0.15 | 0.25 ± 0.19 | 0.26 ± 0.16 |
| **1.12 m.s^-1^** | 0.90 ± 1.17 | 1.08 ± 1.26 | 0.89 ± 1.02 | 0.79 ± 0.72 | 0.89 ± 0.47 | 0.91 ± 0.74 | 0.19 ± 0.20 | 0.17 ± 0.19 | 0.43 ± 0.32 | 0.41 ± 0.30 | 0.42 ± 0.31 | 0.38 ± 0.30 |
| **1.34 m.s^-1^** | 1.11 ± 1.64 | 1.19 ± 1.72 | 1.23 ± 1.42 | 1.05 ± 1.17 | 1.49 ± 0.15 | 1.53 ± 0.90 | 1.16 ± 0.59 | 0.87 ± 0.97 | 0.59 ± 0.35 | 0.62 ± 0.39 | 0.53 ± 0.38 | 0.42 ± 0.41 |
| **3% (0.89 m.s^-1^)** | 0.75 ± 0.91 | 0.90 ± 1.10 | 0.86 ± 0.71 | 0.79 ± 0.59 | 1.40 ± 1.70 | 1.58 ± 1.66 | 0.66 ± 1.36 | 0.61 ± 0.94 | 0.59 ± 0.33 | 0.47 ± 0.33 | 0.44 ± 0.37 | 0.42 ± 0.24 |
| **5% (0.89 m.s^-1^)** | 0.88 ± 1.11 | 1.02 ± 1.29 | 0.75 ± 0.87 | 0.83 ± 0.64 | 0.86 ± 0.52 | 0.79 ± 0.07 | 0.43 ± 0.28 | 0.60 ± 0.41 | 0.79 ± 0.46 | 0.84 ± 0.43 | 0.83 ± 0.47 | 0.82 ± 0.29 |
| **All Activities** | **0.80 ± 0.92** | **0.96 ± 1.00** | **0.76 ± 0.78** | **0.74 ± 0.63** | **1.22 ± 0.93** | **1.06 ± 1.14** | **0.66 ± 0.77** | **0.66 ± 0.62** | **0.42 ± 0.33** | **0.45 ± 0.33** | **0.43 ± 0.35** | **0.39 ± 0.31** |
| **Activity** | **Mean absolute percentage error (%)** | | | | | | | | | | | |
|  | **Unilateral Amputees** | | | | **Bilateral Amputees** | | | | **Control** | | | |
|  | **Longest Limb** | **Spine** | **Shortest Limb** | **Generated model** | **Longest Limb** | **Spine** | **Shortest Limb** | **Generated model** | **Left Limb** | **Spine** | **Right Limb** | **Generated model** |
| **Resting** | - | - | - | - | - | - | - | - | - | - | - | - |
| **0.48 m.s^-1^** | 40 ± 15.5 | 43 ± 24.8 | 36 ± 15.9 | 35 ± 27.1 | 34 ± 18.5 | 40 ± 37.3 | 30 ± 19.9 | 29 ± 18.9 | 23 ± 20.5 | 29 ± 22.5 | 24 ± 23.3 | 15 ± 11.1 |
| **0.67 m.s^-1^** | 26 ± 16.2 | 29 ± 20.2 | 23 ± 12.0 | 22 ± 18.3 | 26 ± 14.6 | 26 ± 23.7 | 19 ± 9.0 | 18 ± 12.6 | 13 ± 14.0 | 12 ± 8.8 | 11 ± 9.7 | 11 ± 10.0 |
| **0.89 m.s^-1^** | 21 ± 17.7 | 25 ± 19.9 | 19 ± 15.0 | 18 ± 12.4 | 26 ± 14.9 | 22 ± 16.7 | 13 ± 9.2 | 13 ± 10.3 | 12 ± 9.4 | 10 ± 5.6 | 11 ± 7.0 | 12 ± 7.6 |
| **1.12 m.s^-1^** | 21 ± 20.2 | 25 ± 20.2 | 20 ± 16.4 | 18 ± 11.8 | 20 ± 16.3 | 25 ± 30.4 | 6 ± 8.3 | 3 ± 2.0 | 16 ± 11.2 | 15 ± 11.1 | 15 ± 11.1 | 14 ± 10.8 |
| **1.34 m.s^-1^** | 17 ± 19.0 | 19 ± 20.7 | 20 ± 16.9 | 18 ± 16.5 | 32 ± 14.2 | 39 ± 38.1 | 22 ± 0.5 | 14 ± 11.2 | 19 ± 12.9 | 20 ± 14.6 | 17 ± 14.0 | 13 ± 13.9 |
| **3% (0.89 m.s^-1^)** | 18 ± 15.7 | 21 ± 18.2 | 20 ± 11.2 | 19 ± 14.2 | 21 ± 17.3 | 25 ± 17.2 | 8 ± 12.5 | 10 ± 10.4 | 13 ± 9.3 | 16 ± 9.6 | 15 ± 10.9 | 15 ± 8.0 |
| **5% (0.89 m.s^-1^)** | 16 ± 14.6 | 18 ± 17.7 | 14 ± 13.3 | 17 ± 12.7 | 14 ± 6.9 | 15 ± 7.3 | 10 ± 9.6 | 14 ± 16.1 | 22 ± 10.8 | 24 ± 9.7 | 23 ± 11.3 | 24 ± 7.3 |
| **All Activities** | **23 ± 18.1** | **26 ± 20.9** | **22 ± 15.2** | **21 ± 17.2** | **26 ± 15.9** | **28 ± 24.9** | **17 ± 14.4** | **16 ± 14.7** | **17 ± 13.2** | **18 ± 13.7** | **17 ± 13.8** | **15 ± 7.3** |
